# Evaluation of Sensititre YeastOne for antifungal susceptibility testing of *Candida (Candidozyma) auris*: misclassification of *FKS1* mutants and overestimation of caspofungin resistance in a global collection of isolates

**DOI:** 10.1128/jcm.00130-26

**Published:** 2026-05-14

**Authors:** Maria Siopi, Sevasti Leventaki, Ioannis Pachoulis, Aikaterini Rentoumi, Nikiforos Argyris, Bram Spruijtenburg, Eelco F. J. Meijer, Jacques F. Meis, Spyros Pournaras, Athanasios Tsakris, Georgia Vrioni, Joseph Meletiadis

**Affiliations:** 1 Clinical Microbiology Laboratory, "Attikon" University General Hospital, Medical School, National and Kapodistrian University of Athens 68993, Athens, Greece; 2 Molecular Microbiology and Immunology Laboratory, Department of Biomedical Sciences, University of West Attica 69104 , Athens, Greece; 3 Department of Medical Microbiology and Ιmmunology, Canisius-Wilhelmina Hospital (CWZ)/Dicoon 6030 , Nijmegen, the Netherlands; 4 Radboudumc-CWZ Center of Expertise for Mycology 519856 , Nijmegen, the Netherlands; 5 Institute of Translational Research, Cologne Excellence Cluster on Cellular Stress Responses in Aging-Associated Diseases (CECAD), Excellence Center for Medical Mycology (ECMM), University of Cologne 14309, Cologne, Germany; 6 Department of Microbiology, Medical School, National and Kapodistrian University of Athens 68993, Athens, Greece; University of Calgary, Calgary, Alberta, Canada

**Keywords:** *Candida (Candidozyma) auris*, sensititre YeastOne, antifungal susceptibility testing

## Abstract

Sensititre YeastOne (SYO) is a widely used commercial colorimetric assay, yet its performance for *C. auris* remains uncertain. We tested 113 global isolates, including 24 *FKS1* mutants, using SYO and the Clinical and Laboratory Standards Institute (CLSI) broth microdilution in parallel. Agreement (±1 twofold dilution) and categorical agreement (CA) were assessed using Centers for Disease Control and Prevention (CDC) tentative resistance breakpoints, CLSI epidemiological cut-off values (ECVs) where available, and the SYO-specific wild-type upper limit value (WT-ULV) for amphotericin B. CLSI-SYO agreement was poor for posaconazole (18%) and amphotericin B (40%); moderate for echinocandins (42%–58%), voriconazole (53%), and itraconazole (65%); and very good for 5-flucytosine (85%). Minimum inhibitory concentrations (MICs) of fluconazole for most isolates were off-scale, precluding accurate estimation. Using CDC breakpoints, amphotericin B showed 31% CA (69% major errors [MaEs]), while fluconazole exhibited 95% CA (4% MaEs, 1% very major errors). Applying the SYO WT-ULV for amphotericin B corrected all discrepancies (100% CA). SYO generated higher MICs than CLSI for 5-flucytosine and azoles, with a resistance-like colorimetric phenotype resulting in high azole MICs in 8% of isolates. Up to 25% of *FKS1* mutants fell below CLSI ECVs by SYO, whereas 14% of WT isolates were overcalled caspofungin-resistant due to a resistance-like colorimetric phenotype. Applying an adjusted interpretive threshold of 0.25 mg/L for anidulafungin and micafungin, and reading growth inhibition endpoints for caspofungin, eliminated the corresponding errors. SYO reliably detects fluconazole and amphotericin B resistance (using the SYO-specific WT-ULV) but frequently misclassifies echinocandin resistance in *C. auris*, particularly for caspofungin and *FKS1* mutants, underscoring the need for careful interpretation and potential method-specific interpretive thresholds.

**IMPORTANCE:**

Accurate antifungal susceptibility testing is critical for guiding antifungal therapy and informing infection prevention and antifungal stewardship strategies for *Candida* (*Candidozyma*) *auris*. As Sensititre YeastOne (SYO) is a widely used commercial colorimetric assay for antifugnal susceptibility testing of *Candida* spp., its performance for *C. auris* was evaluated in 113 global isolates, including 24 *FKS1* mutants. SYO reliably detected fluconazole resistance in *C. auris* at the CDC breakpoint of ≥32 mg/L, while detection of amphotericin B resistance required the SYO-specific wild-type (WT) upper limit value of 8 mg/L. Up to one-quarter of *FKS1* mutants were misclassified as WT, whereas caspofungin resistance was overestimaed in 14% of WT isolates using the CLSI epidemiological cutoff values 1 mg/L for anidulafungin, 0.5 mg/L for micafungin and 0.5 mg/L for caspofungin. Caspofungin should not be used as a standalone marker of echinocandin resistance, while anidulafungin and micafungin consistently detected *FKS1* mutants using the adjusted interpretive threshold of 0.25 mg/L.

## INTRODUCTION

Accurate antifungal susceptibility testing (AFST) is critical for guiding antifungal therapy and informing infection prevention and antifungal stewardship strategies for *Candida* (*Candidozyma*) *auris* (1). This is particularly important given the multidrug resistance observed in some isolates of this species, including the emergence of pan-resistant isolates (2–4). Notably, echinocandins remain first-line agents for invasive candidiasis (5); however, resistance evolving during therapy has been increasingly reported in *C. auris* (6–8), highlighting the need for reliable susceptibility data to enable effective, individualized patient management within the context of limited therapeutic options.

Broth microdilution (BMD) is the reference methodology for AFST of *C. auris* (9, 10), yet its routine implementation is hindered by technical complexity and labor-intensive procedures. Standardized, user-friendly commercial methods that can be readily adopted in diagnostic settings are therefore essential to support timely therapeutic decision-making. The Sensititre YeastOne (SYO) is a ready-to-use, colorimetric AFST assay optimized according to Clinical and Laboratory Standards Institute (CLSI) standards and widely used in clinical laboratories. While acceptable performance has been demonstrated for SYO and common *Candida* species (11), robust data supporting its reliability for *C. auris* are lacking, as prior comparative studies have focused on other commercially available methods (12), relied on potentially clonal isolate collections (12, 13), or small numbers of reference strains (14), and evaluated susceptibility to a single antifungal agent (15). Of note, commercial tests may serve as valid alternatives, provided they demonstrate agreement with the reference method from which the interpretive breakpoints are derived, whereas the known inter-clade variability in *C. auris* susceptibility profiles (16) highlights the need for systematic evaluation using well-characterized and geographically diverse isolates.

Based on these grounds, we assessed SYO performance for AFST of *C. auris* through direct comparison with CLSI BMD, using a collection of isolates encompassing five phylogenetic clades and a range of susceptibility phenotypes, including molecularly verified echinocandin-resistant strains, in an attempt to evaluate the accuracy of SYO-derived minimum inhibitory concentrations (MICs) in a global context.

## RESULTS

### Amphotericin B

**Fig 1:**
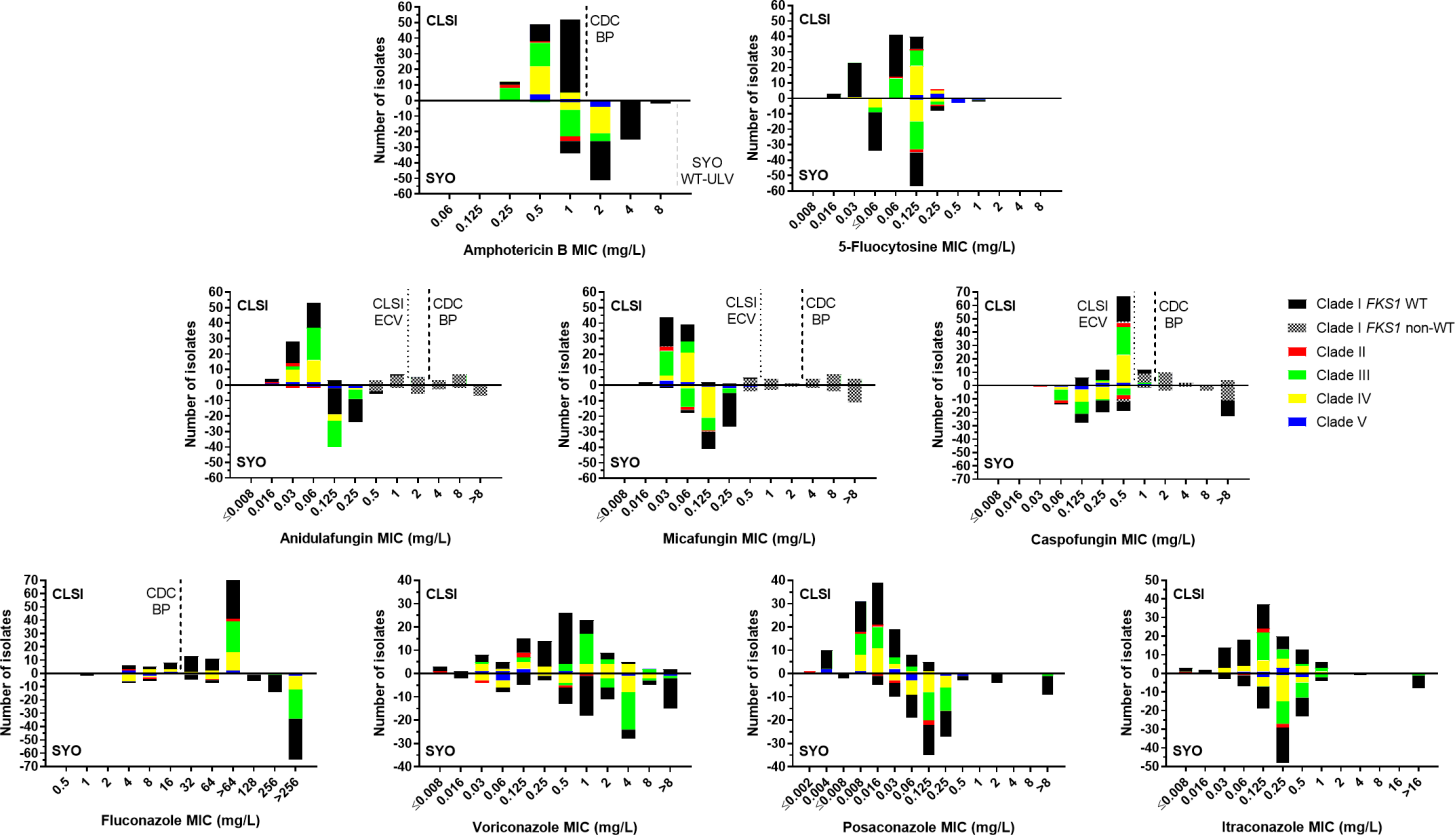
CLSI and SYO MIC distributions stratified by *C. auris* clade. Centers for Disease Control and Prevention (CDC) tentative resistance breakpoints (BPs) (10), CLSI epidemiological cut-off values (ECVs) (17), and the SYO-specific amphotericin B wild-type upper limit value (WT-ULV) (15) are indicated by black broken, black dotted, and gray broken lines, respectively.

Both AFST methods yielded unimodal MIC distributions across all clades; however, CLSI exhibited a narrower range than SYO (3 versus 5 twofold dilutions) and a modal MIC 1 twofold dilution lower (1 versus 2 mg/L) (Fig. 1). CLSI-SYO agreement was poor within ±1 twofold dilution (40%), with a median (range) difference of −2 (0 to −3) twofold dilutions, but increased substantially within ±2 twofold dilutions (95%) (Table 1). A moderate but statistically significant correlation was observed between the two methods (Pearson’s *r* = 0.55, *P* < 0.0001).

**TABLE 1:** Quantitative and qualitative agreement of *C. auris* MIC results generated by CLSI and SYO^*f*^

Drug	CLSI modal (range) MIC (mg/L)	SYO modal (range) MIC (mg/L)	Median (range) difference CLSI-SYO^*a*^	% Agreement		% CA (%MaE, %VmE)
				±1	±2	
Amphotericin B	1 (0.25–1)	2 (1–8)	−2 (−3 to 0)	40	95	31 (69, 0)^*b*^/100 (0, 0)^*c*^
5-Flucytosine	0.06 (0.016–0.25)	0.125 (≤0.06 to 1)	−1 (−3 to 2)	85	98	NA
Anidulafungin	0.06 (0.016 to >8)	0.125 (0.03 to >8)	−1 (−3 to 3)	55	88	96 (3, 1)^*b*^
Micafungin	0.03 (0.016 to >8)	0.125 (0.03 to >8)	−1 (−3 to 3)	58	84	98 (2, 0)^*b*^
Caspofungin	0.5 (0.125 to >8)	0.125 (0.03 to >8)	1 (−7 to 4)	42	68	82 (16, 2)^*b*^/95% (1, 4)^*d*^
Fluconazole	>64 (4 to >64)	>256 (1 to >256)	0 (-2 to 6)^*e*^	52^*e*^	84^*e*^	95 (4, 1)^*b*^
Voriconazole	0.5 (≤0.008 to >8)	4 (0.016 to >8)	−1 (−9 to 2)	53	77	NA
Posaconazole	0.016 (≤0.002 to 0.125)	0.125 (≤0.008 to >8)	−3 (−12 to 2)	18	30	NA
Itraconazole	0.125 (≤0.008 to 1)	0.25 (0.03 to >8)	−1 (−10 to 1)	65	80	NA

^*a*^ Number of twofold dilutions.

^*b*^ Based on the CDC tentative resistance breakpoints (fluconazole ≥32 mg/L; anidulafungin/micafungin ≥4 mg/L; amphotericin B/caspofungin ≥2 mg/L) (10).

^*c*^ Based on the SYO-specific wild-type upper limit value (8 mg/L) (15).

^*d*^ Based on the ~50% growth inhibition endpoint regardless of color change.

^*e*^ Most MICs were off-scale ( >64 mg/L in 70/113 isolates with CLSI; >256 mg/L in 66/113 with SYO). Difference and agreement was calculated only for 25 isolates ( 13 clade I, 1 clade II, 8 clade IV and 3 clade V) with on-scale MICs with both CLSI and SYO methods.

^*f*^ AFST: antifungal susceptibility testing; CA: categorical agreement; MaE: major error; NA: not available; ND: not determined; VmE: very major error.

**Fig 2:**
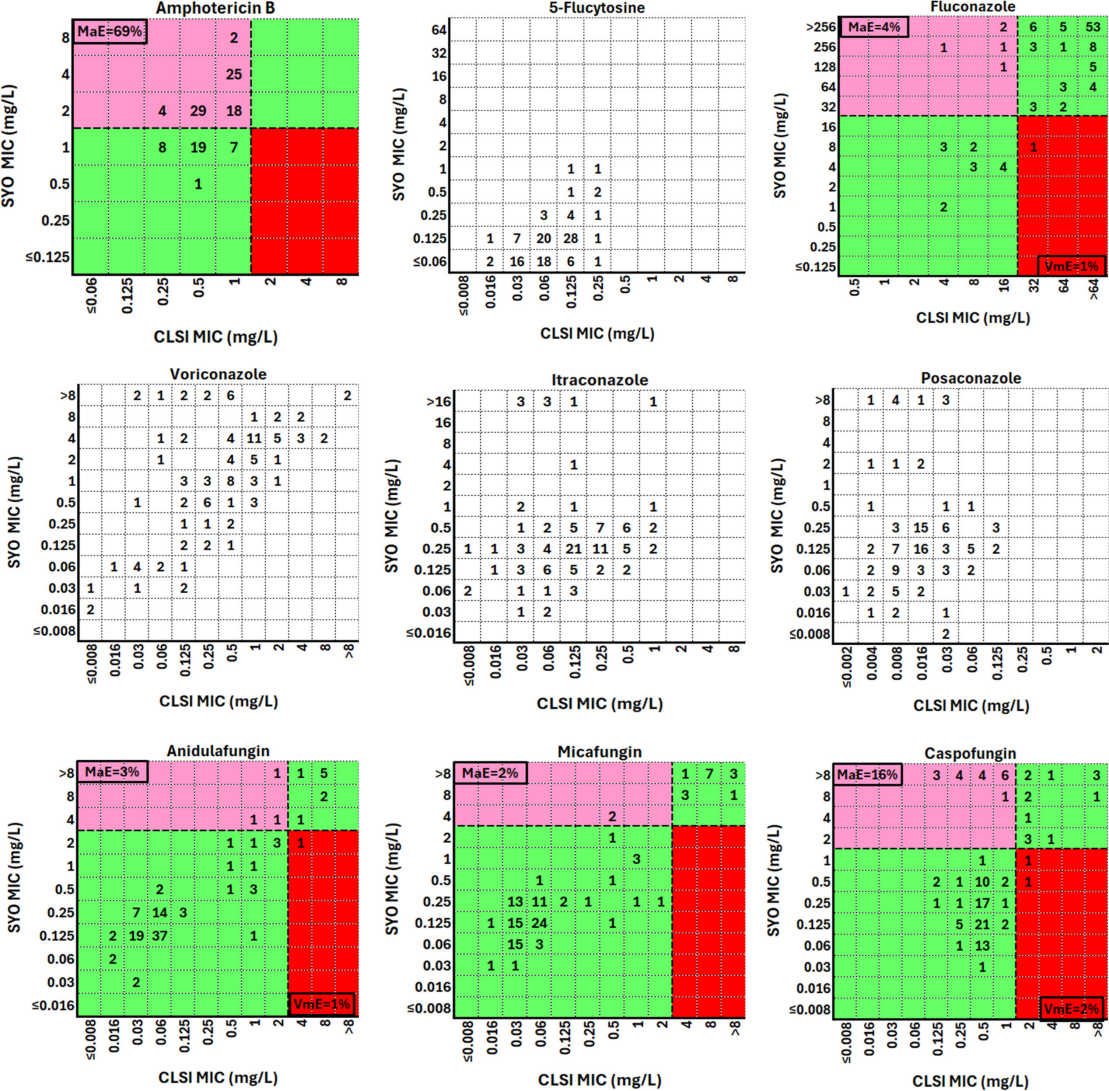
Scatter plots illustrating concordance between CLSI and SYO MIC results. Numbers denote *C. auris* isolates (total *n* = 113) at each MIC pairing. CDC tentative resistance breakpoints for *C. auris* (where available) (10) are indicated by black broken lines. Green shaded areas indicate categorical agreement, whereas pink and red shaded areas represent major error (MaE) and very major error (VmE), respectively.

Using the CDC tentative resistance breakpoint of 2 mg/L, all isolates were classified as non-resistant by CLSI, while 78/113 strains were classified as resistant by SYO, resulting in 31% categorical agreement (CA) and 69% major errors (MaEs) (Cohen’s kappa [*κ*] coefficient = 0; no agreement). On the other hand, when the SYO-specific wild-type upper limit value (WT-ULV) of 8 mg/L was applied, all isolates displayed a WT phenotype based on SYO-derived MICs, yielding complete (100%) CA with CLSI results (Cohen’s *κ* coefficient = 1; perfect agreement) (Fig. 2, Table 1).

### 5-Flucytosine

Both CLSI and SYO MIC distributions were unimodal and spanned five twofold dilutions, with the SYO modal MIC being 1 twofold dilution higher than that of CLSI (0.125 versus 0.06 mg/L) (Fig. 1). CLSI-SYO agreement was very good within ±1 twofold dilution (85%), with a median (range) difference of −1 (−3 to 2) twofold dilution, and further improved within ±2 twofold dilutions (98%). The two AFST methods showed a moderate yet statistically significant correlation (Pearson’s *r* = 0.49, *P* < 0.0001). As no established CLSI MIC interpretive criteria are currently available for resistance detection, CA could not be assessed (Fig. 2, Table 1).

### Echinocandins

For anidulafungin and micafungin, unimodal MIC distributions were recorded for both methods, with CLSI modal MICs being lower than those obtained by SYO by 1 and 2 twofold dilutions, respectively (0.06 and 0.03 versus 0.125 mg/L, respectively). Among *FKS1* WT isolates, MICs clustered at low concentrations by both methods, while isolates harboring *FKS1* mutations exhibited markedly elevated MICs. In particular, CLSI and SYO modal (range) MICs for *FKS1* WT isolates were 0.06 (0.016–1) and 0.125 (0.03–0.5) mg/L for anidulafungin, and 0.03 (0.016–0.5) and 0.125 (0.03–0.5) mg/L for micafungin, compared to 8 (0.5–8) and >8 (0.5 to >8) mg/L for anidulafungin and 8 (0.5 to >8) and >8 (0.5 to >8) mg/L for micafungin for *FKS1* mutants, respectively (Fig. 1).

**Fig 3:**
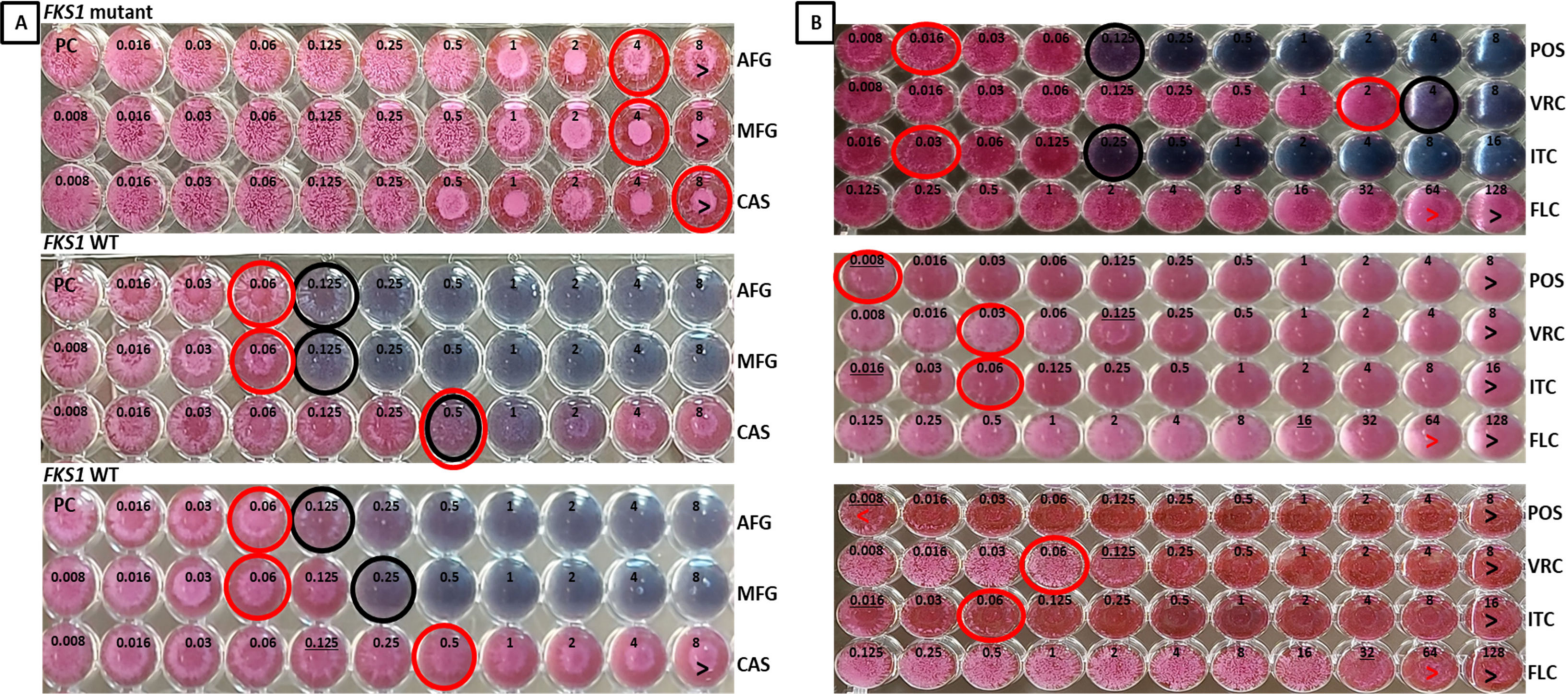
Representative Sensititre YeastOne (SYO) antifungal susceptibility testing patterns in *C. auris*. (**A**) Echinocandin testing showing a *FKS1* mutant with elevated MICs to all echinocandins (upper panel) and two *FKS1* wild-type (WT) isolates exhibiting an Eagle effect (middle) and a resistance-like colorimetric phenotype (lower) for caspofungin. (**B**) Azole testing illustrating isolates without (upper) and with (middle and lower) a resistance-like colorimetric phenotype. Red circles: CLSI MICs. Black circles: SYO MICs defined based on the color change as the first purple or blue well. Red > or <: off-scale CLSI MICs. Black > or <: off-scale SYO MICs. Underlined concentrations: SYO MICs determined using a growth inhibition endpoint corresponding to ~50% reduction in growth, applied only to isolates exhibiting a resistance-like colorimetric phenotype shown. AFG: anidulafungin; CAS: caspofungin; FLC: fluconazole; ITC: itraconazole; MFG: micafungin; PC: positive control; POS: posaconazole; VRC: voriconazole.

For caspofungin, CLSI MICs followed a unimodal distribution with a modal value of 0.5 mg/L, whereas SYO showed a bimodal pattern with two distinct peaks at 0.125 mg/L and >8 mg/L. Among *FKS1* WT isolates, CLSI and SYO modal (range) MICs were 0.5 (0.125–1) mg/L and 0.125 (0.03 to >8) mg/L, respectively. Notably, an Eagle effect was observed in 48/89 (54%) isolates, spanning all clades except clade II and occurring most frequently in clade III (17/23; 74%), followed by clade V (3/5; 60%), clade IV (12/22; 55%), and clade I (16/36; 44%). Moreover, in a subset of clade I isolates (12/36; 33%), pink color persisting across all tested concentrations (“resistance-like colorimetric phenotype”) was observed, yielding high MIC values (>8 mg/L) (Fig. 3). No Eagle effects or resistance-like phenotypes were detected for anidulafungin or micafungin. In contrast, *FKS1* mutants displayed increased MICs by both methods, with CLSI and SYO modal (range) values of 2 (0.5 to >8) mg/L and >8 (0.5 to >8) mg/L, respectively (Fig. 1).

Overall, CLSI-SYO agreement within ±1 twofold dilution was moderate for anidulafungin (55%), micafungin (58%), and caspofungin (42%), with a median (range) difference of −1 (−3 to 3), −1 (−3 to 3), and 1 (−7 to 4) twofold dilution, respectively. Agreement improved when a ±2 twofold dilution criterion was applied, reaching 88% for anidulafungin, 85% for micafungin, and 68% for caspofungin. Correlation between methods was very strong for anidulafungin and micafungin (Pearson’s *r* = 0.90 and 0.92, respectively, *P* < 0.0001), and statistically significant but only weak for caspofungin (Pearson’s *r* = 0.38, *P* < 0.0001) (Table 1).

Using CLSI BMD as the reference, CA was 96% for anidulafungin (3% MaEs, 1% very major errors [VmEs], *κ* = 0.80 [95% CI 0.61–0.99]; strong agreement), 98% for micafungin (2% MaEs, *κ* = 0.93 [95% CI 0.83–1]; almost perfect agreement), and 82% for caspofungin (16% MaEs, 2% VmEs, *κ* = 0.49 [95% CI 0.30–0.67]; weak agreement) (Fig. 2, Table 1). Notably, among *FKS1* mutants, 9/24 (38%), 4/24 (17%), and 1/24 (4%) exhibited CLSI MICs below the corresponding epidemiological cut-off values (ECVs) for anidulafungin, micafungin, and caspofungin, respectively, whereas 5/89 (6%) *FKS1* WT isolates exceeded the caspofungin ECV. When assessed by SYO, 6/24 (25%), 3/24 (12%), and 2/24 (8%) isolates harboring *FKS1* mutations fell below the ECVs for anidulafungin, micafungin, and caspofungin, respectively, while misclassification of *FKS1* WT isolates above the caspofungin ECV was observed in 12/89 (14%) cases. Upon repeat testing of the discrepant isolates, MIC values remained unchanged or within a ±1 twofold dilution range (no change in misclassification rates). Applying an adjusted interpretive threshold of 0.25 mg/L for anidulafungin and micafungin, which is 2 and 1 twofold dilutions lower than current ECVs, respectively, would optimize discrimination between *FKS1* WT and mutant isolates for both CLSI and SYO. This shift would incur only a marginal increase in misclassification of WT isolates: for anidulafungin, 1% for CLSI (one WT isolate with a MIC of 1 mg/L) and 2% for SYO (two WT isolates with MICs of 0.5 mg/L); for micafungin, 1% for both CLSI and SYO (one WT isolate each with MICs of 0.5 mg/L). Subsequent testing of the discrepant isolates yielded MICs that were identical or within a ±1 twofold dilution (no impact on misclassification rates). On the other hand, no interpretive threshold adjustment was able to improve the performance of either method for caspofungin (Fig. 1, Table 2).

**TABLE 2:** CLSI and SYO echinocandin susceptibility profiles of 24 clade I *C. auris* isolates harboring *FKS1* mutations^*a*^

ID	*FKS1* genotype	Anidulafungin MIC (mg/L)		Micafungin MIC (mg/L)		Caspofungin MIC (mg/L)	
		CLSI	SYO	CLSI	SYO	CLSI	SYO
AMU138	F635Y	1	0.5	2	0.5	2	0.5
AMU139	F635Y	1	0.5	1	0.5	1	0.5
AUH4078	F635Y	1	1	1	1	1	>8
AMU129	S639F	4	4	8	>8	2	1
AMU134	S639F	8	>8	8	>8	2	4
AMU135	S639F	8	>8	8	>8	2	>8
AMU140	S639F	0.5	2	0.5	4	1	>8
AMU141	S639F	2	>8	8	>8	1	8
AMU144	S639F	0.5	1	0.5	2	0.5	1
AMU145	S639F	2	2	4	8	4	2
AMU146	S639F	2	2	4	8	2	2
AMU147	S639F	1	2	4	8	2	2
AMU128	S639P	1	4	8	>8	2	2
AMU132	S639P	8	>8	8	>8	4	>8
AMU111	S639T	2	2	1	1	1	>8
AUH2689	S639Y	4	>8	4	>8	8	>8
AMU133	S639Y	2	4	0.5	4	1	>8
AMU142	M690I	1	0.5	1	1	1	>8
AMU137	M690V	8	8	8	>8	2	8
AUH4077	M690V	0.5	0.5	0.5	0.5	2	8
AMU130	Δ635F	8	>8	>8	>8	>8	>8
AMU131	Δ635F	8	>8	>8	>8	>8	>8
AMU143	Δ635F	4	2	>8	>8	>8	>8
AUH2883	Δ635F	8	8	>8	8	>8	8
No of non-resistant isolates (%)		14 (58%)	12 (50%)	9 (38%)	7 (29%)	8 (33%)	4 (17%)
No of WT isolates (%)		9 (38%)	6 (25%)	4 (17%)	3 (12%)	1 (4%)	2 (8%)

^*a*^ Shaded cells indicate non-resistance according to the CDC tentative resistance breakpoints (anidulafungin/micafungin/caspofungin ≥4/≥4/≥2 mg/L) (10). Underlined values indicate WT phenotype based on CLSI ECVs (anidulafungin/micafungin/caspofungin ≤1/≤0.5/≤0.5 mg/L) (17). AMU, Attikon Mycology Unit; AUH, Attikon University Hospital.

Nonetheless, among the 12 *FKS1* WT isolates exhibiting a resistance-like colorimetric phenotype for caspofungin, on-scale MICs could be determined by applying a growth inhibition endpoint corresponding to a prominent reduction of growth (~50%) in SYO (Fig. 3), increasing agreement with CLSI to 75%/83% within ±1/±2 twofold dilutions, with 11/12 now correctly classified as WT. When this endpoint was applied to all isolates, overall CA increased to 95% (1% MaEs, 4% VmEs), despite a small reduction in agreement to 30%/65% within ±1/±2 twofold dilutions, as growth-based MICs were higher than colorimetric MICs. For anidulafungin and micafungin, application of the same endpoint increased agreement to 76%/97% and 85%/96% within ±1/±2 twofold dilutions, respectively, but reduced CA to 88% and 96%, with corresponding VmEs of 12% and 4%.

### Azoles

MIC distributions obtained by both CLSI and SYO were broad, spanning ≥6 to ≥12 twofold dilutions, although the precise number of dilution steps could not be ascertained due to off-scale values, particularly for SYO. According to CLSI, itraconazole and posaconazole displayed unimodal distributions with modal MICs of 0.125 mg/L and 0.016 mg/L, respectively, as opposed to voriconazole, which exhibited a trimodal pattern (0.03, 0.125, and 0.5 mg/L). In contrast, SYO yielded bimodal distributions for itraconazole (0.25 and >16 mg/L) and posaconazole (0.125 and >8 mg/L), and a multimodal distribution for voriconazole (0.06, 1, 4, and >8 mg/L). Evaluation of fluconazole MIC distribution peaks was precluded by the high proportion of off-scale values (CLSI: >64 mg/L in 70/113 isolates; SYO: >256 mg/L in 66/113) (Fig. 1).

In total, CLSI-SYO agreement within ±1 twofold dilution was moderate for fluconazole (52% for onscale MICs), voriconazole (53%) and itraconazole (65%), but poor for posaconazole (18%), with a median difference of 0 (-2 to 6) , −1 (−9 to 2), −1 (−10 to 2), and −3 (−12 to 2) twofold dilutions, respectively. Higher agreement was found within ±2 twofold dilutions (posaconazole 30%, voriconazole 77%, and itraconazole 80%, fluconazole 84%) (Table 1). Voriconazole and onscale fluconazole MIC values generated with the two methods showed statistically significant but moderate correlation (Pearson’s *r* = 0.58 and 0.59, respectively, *P* < 0.002), as opposed to posaconazole and itraconazole MICs that were not correlated (Pearson’s *r* = 0.04 and 0.06, respectively, *P* ≥ 0.56).

According to CLSI-derived MICs, most isolates were classified as fluconazole-resistant (94/113; 83%), with marked clade-specific variability (83% in clade I, 67% in clade II, 100% in clade III, 77% in clade IV, and 40% in clade V). CA for fluconazole was 95% (4% MaEs, 1% VmEs, *κ* = 0.79 [95% CI 0.63–0.95]; moderate agreement). As CDC tentative interpretive criteria are available only for fluconazole, which serves as a surrogate for the azole class, CA could not be evaluated for the remaining azoles (Fig. 2, Table 1).

Of note, a resistance-like colorimetric phenotype was observed in 9/113 (8%) isolates, predominantly from clade I (8/60; 13%) and one from clade III (1/23; 4%), characterized by minimal visible growth across the dilution range but uniform conversion of all wells to pink, resulting in extreme SYO MIC values for all three azoles (itraconazole >16 mg/L, posaconazole >8 mg/L, and voriconazole >8 mg/L) (Fig. 3). When growth rather than color was evaluated to determine SYO MICs for these isolates, application of a ~50% growth inhibition endpoint restored concordance with CLSI results. Specifically, agreement within ±1/±2 twofold dilutions increased from 0% for all agents to 38%/75% for itraconazole, 63%/100% for posaconazole, and 25%/63% for voriconazole. When the same endpoint was applied to all isolates, improved agreement was maintained for posaconazole (65%/87%) and voriconazole (60%/80%), but not for itraconazole (51%/75%). In contrast, for fluconazole, growth inhibition MICs were lower than colorimetric MICs. Among isolates with a resistance-like colorimetric phenotype, this led to a significant reduction in CA from 89% to 44% and a concomitant rise in VmEs from 11% to 56%. When extended to all isolates, CA declined from 95% (4% MaE, 1% VmE) with colorimetric MICs to 80% (1% MaE, 19% VmEs) using growth-based MICs.

### Reproducibility

Absolute inter-observer agreement was excellent for both CLSI and SYO (96% and 95%, respectively, 100% within ±1 twofold dilution), with quality control MIC values falling within the expected ranges for both methods. Absolute inter-experimental agreement for SYO colorimetric endpoints was 68%, increasing to 96% within ±1 twofold dilution, while CA between blinded readers and across independent replicates was excellent (100%).

## DISCUSSION

Given the widespread clinical reliance on commercial AFST methods for managing *C. auris* infections, a systematic comparison of the SYO assay with the CLSI reference BMD method across an international panel of isolates revealed marked, drug-specific discrepancies. Based on CDC breakpoints, SYO accurately identified fluconazole resistance (95% CA, 4% MaEs, and 1% VmEs), while performance for amphotericin B was poor (31% CA and 69% MaEs) unless interpretation was based on the SYO-specific WT-ULV of 8 mg/L (15), which restored complete CA. Overall CA appeared high for echinocandins (82%–98%), yet up to one-quarter with SYO and one-third with CLSI of *FKS1* mutants fell below CLSI ECVs. Caspofungin resistance was overestimated in 14% of WT strains due to a resistance-like colorimetric phenotype (pink color across all concentrations), which could be overcome by determining a ~50% growth inhibition endpoint regardless of color (CA 95%, 1% MaEs, and 4% VmEs). Applying an adjusted interpretive threshold of 0.25 mg/L for anidulafungin and micafungin improved discrimination between *FKS1* WT and mutant isolates (100% CA), effectively capturing mutants with MICs at or below the current CLSI ECVs, while maintaining low misclassification rates for WT isolates (1%–2%). SYO yielded higher MICs than CLSI for 5-flucytosine and azoles, with a resistance-like colorimetric phenotype producing extreme azole MICs in a subset of isolates (8%). For these isolates, growth rather than color assessment improved agreement with CLSI, but only for posaconazole and voriconazole.

The reliability of AFST for *C. auris* has direct clinical and public health implications given its potential for a multidrug-resistant phenotype and limited treatment options. To our knowledge, this is the first comprehensive evaluation of SYO performance using a phylogenetically and geographically diverse panel of *C. auris* isolates, including genotypically confirmed *FKS1* mutants. Direct comparison with the CLSI BMD methodology strengthens the interpretive framework and addresses gaps in previous investigations, which either lacked reference alignment (12), focused on potentially clonal Indian clade I (12) or Spanish clade III (13) isolates, were restricted to a small set of reference strains (14), did not assess molecularly characterized echinocandin-resistant strains (12–14), compared SYO solely with European Committee on Antimicrobial Susceptibility Testing (EUCAST) BMD method (13), or did not perform AFST by the two methods in parallel (13, 14). These considerations are particularly relevant given the documented inter-clade variability in *C. auris* antifungal susceptibility (16). Studies are usually conducted using predominantly non-resistant/WT isolates, and therefore, they are unable to assess VmEs (18). Moreover, SYO performance has been optimized with CLSI criteria rather than EUCAST, and comparative assessments of commercial assays should ensure alignment with the standards underlying their interpretive criteria. It should also be noted that performing MIC determinations concurrently across methods maintains consistency and minimizes variation attributable to inoculum size or incubation conditions. Together, these design features enhance the robustness, generalizability, and clinical relevance of our findings, offering a solid basis for interpreting SYO-derived susceptibility data in routine laboratory practice across the global diversity of *C. auris*.

AFST of amphotericin B against *C. auris* using commercially available methods has raised early concern, prompting both CLSI and EUCAST to issue warnings against their routine use (19, 20). This is clinically significant given the role of liposomal amphotericin B as a second-line therapeutic option (21), particularly amid the increasing prevalence of echinocandin-resistant infections (6–8). Several commercial platforms, including the Vitek 2 system and gradient concentration strips, have consistently shown poor and moderate CA with the CLSI BMD, respectively, when adopting the CDC breakpoint of 2 mg/L, necessitating method-specific interpretive criteria to achieve acceptable performance (22–24). In contrast, the Micronaut-AM assay has demonstrated more favorable results; nonetheless, these evaluations were constrained by the absence of the gold standard BMD method as a comparator and by reliance on potentially clonal (25) or genetically identical (26) clade I isolates, which may underestimate the range of MIC variability encountered in real-world laboratory settings. With respect to SYO, the quantitatively expanded panel of isolates used in the present study confirms prior observations (15) of resistance overestimation (≥2 mg/L; 31% CA, 69% MaEs) and reinforces the need for a SYO-specific interpretive threshold (WT-ULV ≤8 mg/L; 100% CA), a requirement that has already been proposed for other *Candida* species (27). While isolates with defined resistance mechanisms were not included in the CLSI-SYO comparison, sporadic cases of clinically acquired amphotericin B resistance have been reported (SYO MICs not available, Etest MICs >32 mg/L) (28, 29), whereas amphotericin B resistance in *C. auris* may be inducible and transient, with MICs decreasing following *in vitro* passages under drug-free conditions (30).

Regarding 5-flucytosine, SYO-derived MICs displayed a unimodal distribution and exceeded those of CLSI while showing strong quantitative agreement (85%/98% within ±1/±2 twofold dilutions). The modal SYO MIC observed aligns with previous reports (0.125 mg/L) (31); however, as this study represents the first head-to-head evaluation of SYO and CLSI for this agent in *C. auris*, the results cannot be directly compared with other data, yet they provide valuable reference for future assessments. Of note, other commercial AFST assays, such as the Vitek 2 system and MIC test strips, have shown minimal to poor agreement with CLSI BMD methodology (0%/3% and 37%/69% within ±1/±2 twofold dilutions, respectively) (22, 24). While 5-flucytosine currently plays a limited role in managing *C. auris* infections (21), its incorporation into antifungal combination therapy is increasingly considered (32), particularly in the context of emerging multidrug resistance. To date, only a limited number of resistant clinical isolates have been described, carrying mutations in *ADE17* + *FUR1*, *CrcB +FCY2,* and *FUR1*, with SYO MICs ≥ 64 mg/L (4, 33), underscoring the potential for emerging resistance and the importance of ongoing surveillance.

For anidulafungin and micafungin, MIC distributions obtained by both CLSI and SYO were unimodal, with low values observed among *FKS1* WT isolates and elevated values among *FKS1* mutants. In contrast, caspofungin MICs followed a unimodal pattern by CLSI, as opposed to SYO, which exhibited a bimodal distribution reflecting the occurrence of a resistance-like colorimetric phenotype in 14% of *FKS1* WT isolates (affecting one-third of clade I strains) that led to extreme MICs (>8 mg/L), while *FKS1* mutants had uniformly high SYO MICs (0.5 to >8 mg/L). This phenotype is believed to represent a fully developed Eagle effect, which typically begins as a classical Eagle effect (pink color in only 2–3 wells at high concentrations) around 21–22 h of incubation and progresses to pink coloration across all concentrations by 24 h, as observed in 70% of clonal clade I Indian isolates (12). In fact, *FKS1* WT strains can display variable inhibition patterns in the presence of increasing caspofungin concentrations, yet paradoxical growth does not translate into poor *in vivo* response (34). Notably, our results indicate that using a ~50% growth inhibition endpoint in SYO largely corrected the misclassification of WT isolates with a resistance-like colorimetric phenotype, increasing CA from 0% to 92%, suggesting that such an approach may be useful to address this phenotypic pattern. No such effects have been observed for anidulafungin or micafungin with SYO (12), corroborating our findings. Overall, CLSI-SYO MIC agreement was moderate for all three echinocandins (42%–58%).

Nonetheless, since SYO is optimized and marketed strictly as a colorimetric assay, adherence to the manufacturer’s instructions remains the standard for routine laboratory practice. While visual assessment of growth may provide clarity in the presence of a resistance-like colorimetric phenotype, deviating from the colorimetric endpoint should be approached with caution and may be best reserved as a supplemental troubleshooting step for these instances. Despite the potential for a growth-based endpoint to reduce the high rate of MaEs observed in certain clades, this “off-label” use carries limitations that should be acknowledged. Specifically, it introduces subjectivity into the interpretation, may increase the risk of VmEs, as observed in our fluconazole data, and complicates standardized laboratory validation.

Although the CLSI-SYO CA was high for anidulafungin (96%; 3% MaEs, 1% VmEs) and micafungin (98%; 2% MaEs), the current CLSI ECVs (1 and 0.5 mg/L, respectively) (17) bisected the mutant MIC distributions for both methods. As a result, up to 25% of isolates carrying *FKS1* mutations were classified as WT by both CLSI and SYO. Notably, *FKS1* mutants with anidulafungin CLSI MICs of 1 mg/L (F635Y) (35) and SYO MICs of 0.5–1 (S639P, D642Y) (31), as well as micafungin CLSI MICs of 0.5 mg/L (F635L) (35) and SYO MICs of 0.5 mg/L (D642Y) (31), have been reported. In this context, applying an adjusted interpretive threshold of 0.25 mg/L would optimize the detection of *FKS1*-mediated resistance with minimal impact on MaE rates (1%–2%). Thus, isolates with MICs 1 twofold dilution above these proposed thresholds (i.e., 0.5 mg/L) may warrant confirmatory testing using gradient concentration strips, which have shown superior discriminatory ability compared with CLSI BMD (22). Interestingly, these method-adjusted interpretive thresholds align with the recently established EUCAST clinical breakpoint for both agents (9). On the other hand, no interpretive threshold adjustment could overcome caspofungin-related discrepancies, underscoring its limited reliability as a marker of echinocandin resistance in *C. auris*, as observed in other species (36). Until outcome-driven breakpoints become available, micafungin appears better suited as a surrogate indicator of echinocandin resistance in *C. auris* when testing by SYO, supported by stronger inter-method concordance and better correlation with *FKS1* status, consistent with observations in common *Candida* species (37).

These findings have important clinical implications given that echinocandins remain first-line agents for the treatment of invasive candidiasis (5), whereas resistance mediated by *FKS1* mutations has been increasingly reported in *C. auris* (6–8). Misclassification of isolates with MICs near current ECVs raises concern that reliance on phenotypic susceptibility results alone may fail to detect emerging resistance, particularly in the setting of prior echinocandin exposure or clinical non-response. In such scenarios, confirmatory AFST or molecular analysis of *FKS1* hotspot regions should be considered. From an antifungal stewardship perspective, inaccurate susceptibility categorization may lead to inappropriate continuation of echinocandin therapy, thereby increasing the risk of treatment failure and further selection of resistant strains. These considerations are particularly relevant for laboratories relying exclusively on SYO for echinocandin susceptibility testing, highlighting the need for heightened awareness of method-specific limitations and for integrating MIC results within the broader clinical and, where available, molecular context.

Fluconazole is a key reference agent for azole susceptibility testing in *C. auris* since interpretive criteria are currently available only for this compound, which is used as a surrogate for the azole class (10). MIC distributions generated by both CLSI and SYO were dominated by off-scale high values, consistent with previous SYO reports (12, 13, 31, 38), thereby precluding detailed distributional analysis and underscoring the high prevalence of resistance across the collection, along with interclade variability. Of note, persistent *C. auris* infection despite standard and high-dose fluconazole therapy (400–800 mg/day), caused by a fluconazole-non-resistant isolate with a CLSI MIC of 8 mg/L, has been documented (39), raising concerns regarding the clinical relevance of the CDC breakpoint of 32 mg/L (10). However, in that case, a central venous catheter remained in place for most of the treatment duration (39), which may have contributed to persistent positivity regardless of the antifungal’s efficacy. In this context, the primary requirement for fluconazole AFST is accurate and reproducible discrimination between resistant and non-resistant isolates rather than fine-scale MIC resolution. Performance among currently available commercial AFST assays varies; while gradient concentration strips achieved high CA with CLSI (98%–100%) (22, 40), Vitek 2 (24, 40), and Micronaut-AM (26) were associated with high VmE rates (31%–32% and 6%, respectively) and underestimation of resistance. In the present study, SYO demonstrated high CA with CLSI (95% CA, 4% MaEs, and 1% VmEs), as previously shown with EUCAST (100% CA) (13), supporting its utility for fluconazole resistance detection in *C. auris*, albeit careful interpretation remains warranted given the complex relationship between *in vitro* fluconazole non-resistance and clinical outcome.

For the remaining azoles, both CLSI and SYO yielded broad MIC distributions spanning multiple twofold dilutions. SYO MICs displayed bimodal or multimodal patterns for itraconazole, posaconazole, and voriconazole, in line with the heterogeneous susceptibility profiles previously described for *C. auris* (12, 38). CLSI-SYO quantitative agreement was drug-dependent and overall limited, being moderate for itraconazole (65%) and voriconazole (53%), and poor for posaconazole (18%). Similar inconsistencies have been described for other commercial AFST assays, including Vitek 2 and gradient concentration strips, which also demonstrated wide MIC ranges and variable, drug-specific concordance with the CLSI BMD method (13, 22, 24, 40), underscoring the lack of harmonization in azole susceptibility testing for *C. auris*. In the absence of established interpretive criteria for these agents, the clinical relevance of MIC variability remains uncertain (41). Notably, uniformly off-scale SYO MICs across all azoles were observed in a subset of isolates (8%), predominantly within clade I (13%), highlighting challenges inherent to colorimetric endpoint interpretation. Similar discrepancies, characterized by extreme SYO MICs relative to substantially lower CLSI values (>8 and >8 mg/L for SYO versus 0.03 and 0.25 mg/L for CLSI), have been reported previously (42). A careful inspection of the well bottoms indicated that the pink coloration noted was not associated with heavy growth. Indeed, when SYO MICs were determined for the resistance-like colorimetric phenotype isolates based on ~50% growth inhibition, ignoring the color change, concordance with CLSI MICs was improved. Given the high fluconazole MICs even in isolates with low MICs to the other azoles, cross-resistance in the majority of the isolates cannot be ruled out. Together, these findings indicate that susceptibility results for azoles other than fluconazole should be interpreted cautiously and are currently better suited for epidemiological surveillance than for guiding antifungal therapy.

It should be noted, however, that the present study was conducted in a single laboratory, precluding the assessment of inter-laboratory variability for both the CLSI and SYO methods. Further multi-center evaluations are warranted to confirm the generalizability of these drug-specific discrepancies and the proposed interpretive adjustments across different settings.

In conclusion, SYO reliably detected fluconazole resistance in *C. auris* at the CDC breakpoint of ≥32 mg/L, while detection of amphotericin B resistance required the SYO-specific WT-ULV of ≤8 mg/L. Caspofungin should not be used as a standalone marker of echinocandin resistance, whereas anidulafungin and micafungin consistently detect *FKS1* mutants when assessed using the adjusted interpretive threshold of 0.25 mg/L. Awareness of these parameters is crucial for laboratories reporting SYO results, particularly in settings with a high prevalence of echinocandin resistance. These findings highlight the critical need for drug- and method-specific interpretive criteria to ensure accurate AFST and to guide clinical and surveillance decision-making in *C. auris*.

## MATERIALS AND METHODS

### Isolates

A total of 113 *C. auris* isolates were tested. Of these, 30 were collected between June 2021 and September 2025 from individual patients admitted to 17 Greek tertiary care hospitals. Species-level identification was performed using matrix-assisted laser desorption ionization-time of flight mass spectrometry (Bruker Daltonics, Bremen, Germany), and all isolates were assigned to clade I (43). An additional 83 genetically distinct isolates representing five *C. auris* clades, sourced from diverse geographic regions, were also included. These comprised *n* = 30 clade I (South Asian; Brazil, Kuwait, Iran, India, Oman, Pakistan), *n* = 3 clade II (East Asian; South Korea, Japan), *n* = 23 clade III (African; South Africa, Spain), *n* = 22 clade IV (South American; Venezuela, Colombia), and *n* = 5 clade V (Iranian; Iran) isolates (43). Among the collection, *n* = 24 clade I isolates carried *FKS1* hotspot mutations, either non-synonymous substitutions (F635Y, S639F/P/T/Y, M690V/I) or a deletion (ΔF635), which are known to confer resistance/reduced susceptibility to echinocandins and were detected as previously described (35).

All isolates were stored at −70°C in sterile normal saline with 10% glycerol (AppliChem, Darmstadt, Germany) until use. Prior to testing, they were revived by two consecutive subcultures on in-house prepared, antimicrobial-free Sabouraud dextrose agar (Oxoid, Athens, Greece) at 35 ± 2°C for 24 h.

### AFST

CLSI BMD AFST was carried out according to the M27-A4 protocol using laboratory-grade pure powders of amphotericin B, fluconazole, itraconazole, posaconazole, 5-flucytosine (all from Sigma-Aldrich, Athens, Greece), voriconazole (Pfizer Ltd., Kent, UK), anidulafungin (Pfizer, CT, USA), caspofungin (Merck & Co., NJ, USA), and micafungin (Astellas Pharma, Tokyo, Japan). Microtitration plates were incubated at 35°C ± 2°C in ambient air for 24 h, and MICs were defined as the lowest drug concentration resulting in a prominent reduction (~50%) in visual growth for azoles, echinocandins, and 5-flucytosine, or complete growth inhibition for amphotericin B, compared with the drug-free growth control (44).

SYO AFST was performed in accordance with the manufacturer’s recommendations using the YO10 panel (Thermo Fisher Scientific, Waltham, MA, USA). Plates were incubated at 35°C for 24 h, and colorimetric MICs were recorded as the first purple or blue well for azoles, echinocandins, and 5-flucytosine, or the first blue well for amphotericin B, provided that the well containing the drug-free growth control was pink.

All isolates were tested in parallel using both AFST methods with a single yeast suspension prepared in sterile distilled water and adjusted to the appropriate inoculum density. Inoculum concentration and purity were verified by spread plating on in-house prepared, antimicrobial-free Sabouraud dextrose agar. MIC endpoints were read visually and independently by two blinded observers under standard laboratory lighting with the aid of a magnifying mirror; discrepancies were resolved by a third reader. Isolates yielding discordant MIC results between methods were re-tested using both CLSI BMD and SYO, and results obtained upon repeat testing were considered definitive. *Pichia kudriavzevii* (syn. *C. krusei*) ATCC 6258 and *C. parapsilosis* ATCC 22019 were included as quality control strains for both methodologies. To assess inter-day reproducibility of SYO MIC determination, 14/113 isolates were re-tested on different days.

### Analysis

A head-to-head comparison of MIC data sets generated by the two AFST methods was conducted, with CLSI BMD serving as the reference standard. High off-scale MIC values were rounded up to the next highest twofold concentration, whereas low off-scale values were retained as recorded. For quantitative analysis, MICs were converted to a log₂ scale. Pearson correlation analysis was used to evaluate the relationship between CLSI and SYO MICs for each antifungal agent, and a two-tailed *P* value < 0.05 was considered statistically significant. Absolute inter-observer agreement and inter-day reproducibility were assessed as the proportion of isolates with identical MICs. Agreement between methods was determined by calculating the proportion of isolates with MICs within ±1 and ±2 twofold dilutions. For qualitative analysis, CA was assessed using the CDC tentative resistance breakpoints (fluconazole ≥32 mg/L; anidulafungin and micafungin ≥4 mg/L; amphotericin B and caspofungin ≥2 mg/L) (10), as well as the SYO-specific WT-ULV for amphotericin B (8 mg/L) (15). The strength of CA was quantified using Cohen’s *κ* coefficient. Discrepancies were defined as MaE when an isolate was categorized as non-resistant by CLSI but resistant by SYO, and as VmE when an isolate was classified as resistant by CLSI but non-resistant by SYO. The ability of each method to identify *FKS1* mutants was further evaluated using the corresponding CLSI ECVs for anidulafungin (1 mg/L) and caspofungin/micafungin (0.5 mg/L) (17). Statistical analyses were performed using GraphPad Prism software, version 9.0 for Windows (GraphPad Software, San Diego, CA, USA).

## References

[1] Dennis EK, Chaturvedi S, Chaturvedi V. 2021. So many diagnostic tests, so little time: review and preview of Candida auris testing in clinical and public health laboratories. Front Microbiol 12:757835. doi:10.3389/fmicb.2021.757835 34691009 PMC8529189

[2] Kim HY PhD, Nguyen TA MSc, Kidd S PhD, Chambers J MD, Alastruey-Izquierdo A PhD, Shin J-H MD, Dao A PhD, Forastiero A MD, Wahyuningsih R MD, Chakrabarti A MD, Beyer P, Gigante V PhD, Beardsley J PhD, Sati H PhD, Morrissey CO PhD, Alffenaar J-W PhD. 2024. Candida auris-a systematic review to inform the world health organization fungal priority pathogens list. Med Mycol 62:42. doi:10.1093/mmy/myae042 PMC11210622 38935900

[3] Ben Abid F, Salah H, Sundararaju S, Dalil L, Abdelwahab AH, Salameh S, Ibrahim EB, Almaslmani MA, Tang P, Perez-Lopez A, Tsui CKM. 2023. Molecular characterization of Candida auris outbreak isolates in Qatar from patients with COVID-19 reveals the emergence of isolates resistant to three classes of antifungal drugs. Clin Microbiol Infect 29:1083. doi:10.1016/j.cmi.2023.04.025 PMC10132836 37116861

[4] Jacobs SE, Jacobs JL, Dennis EK, Taimur S, Rana M, Patel D, Gitman M, Patel G, Schaefer S, Iyer K, Moon J, Adams V, Lerner P, Walsh TJ, Zhu YC, Anower MR, Vaidya MM, Chaturvedi S, Chaturvedi V. 2022. Candida auris pan-drug-resistant to four classes of antifungal agents. Antimicrob Agents Chemother 66:e0005322. doi:10.1128/aac.00053-22 35770999 PMC9295560

[5] Cornely OA, Sprute R, Bassetti M, Chen SC-A, Groll AH, Kurzai O, Lass-Flörl C, Ostrosky-Zeichner L, Rautemaa-Richardson R, Revathi G, et al.. 2025. Global guideline for the diagnosis and management of candidiasis: an initiative of the ECMM in cooperation with ISHAM and ASM. Lancet Infect Dis 25:e280–e293. doi:10.1016/S1473-3099(24)00749-7 39956121

[6] Meletiadis J, Siopi M, Spruijtenburg B, Georgiou P-C, Kostoula M, Vourli S, Frantzeskaki F, Paramythiotou E, Meis JF, Tsangaris I, Pournaras S. 2024. Candida auris fungaemia outbreak in a tertiary care academic hospital and emergence of a pan-echinocandin resistant isolate, Greece, 2021 to 2023. Euro Surveill 29:2400128. doi:10.2807/1560-7917.ES.2024.29.45.2400128 39512169 PMC11544718

[7] Codda G, Willison E, Magnasco L, Morici P, Giacobbe DR, Mencacci A, Marini D, Mikulska M, Bassetti M, Marchese A, Di Pilato V. 2023. In vivo evolution to echinocandin resistance and increasing clonal heterogeneity in Candida auris during a difficult-to-control hospital outbreak, Italy, 2019 to 2022. Euro Surveill 28:2300161. doi:10.2807/1560-7917.ES.2023.28.14.2300161 37022211 PMC10283462

[8] Spruijtenburg B, Ahmad S, Asadzadeh M, Alfouzan W, Al-Obaid I, Mokaddas E, Meijer EFJ, Meis JF, de Groot T. 2023. Whole genome sequencing analysis demonstrates therapy-induced echinocandin resistance in Candida auris isolates. Mycoses 66:1079–1086. doi:10.1111/myc.13655 37712885

[9] Arendrup MC, Guinea J, Arikan-Akdagli S, Meijer EFJ, Meis JF, Buil JB, Dannaoui E, Giske CG, Lyskova P, Meletiadis J, Subcommittee on Antifungal Susceptibility Testing of the ESCMID European Committee for Antimicrobial Susceptibility Testing. 2026. How to interpret MICs of amphotericin B, echinocandins and flucytosine against Candida auris (Candidozyma auris) according to the newly established European Committee for Antimicrobial Susceptibility Testing (EUCAST) breakpoints. Clin Microbiol Infect 32:56–61. doi:10.1016/j.cmi.2025.07.002 40651666

[10] CDC. 2024. Antifungal Susceptibility Testing for C. auris. https://www.cdc.gov/candida-auris/hcp/laboratories/antifungal-susceptibility-testing.html.

[11] Kidd SE, Crawford LC, Halliday CL. 2021. Antifungal susceptibility testing and identification. Infect Dis Clin North Am 35:313–339. doi:10.1016/j.idc.2021.03.004 34016280

[12] Patwardhan SA, Prayag PS, Soman RN, Purandare BD, Ramya S, Dawra R, Joshi R, Prayag AP. 2024. Candida auris - Comparison of sensititre YeastOne and Vitek 2 AST systems for antifungal susceptibility testing - A single centre experience. Indian J Med Microbiol 50:100618. doi:10.1016/j.ijmmb.2024.100618 38795936

[13] Ruiz-Gaitán AC, Cantón E, Fernández-Rivero ME, Ramírez P, Pemán J. 2019. Outbreak of Candida auris in Spain: A comparison of antifungal activity by three methods with published data. Int J Antimicrob Agents 53:541–546. doi:10.1016/j.ijantimicag.2019.02.005 30769198

[14] Ceballos-Garzon A, Holzapfel M, Welsch J, Mercer D. 2025. Identification and antifungal susceptibility patterns of reference yeast strains to novel and conventional agents: a comparative study using CLSI, EUCAST and Sensititre YeastOne methods. JAC Antimicrob Resist 7:dlaf040. doi:10.1093/jacamr/dlaf040 40110552 PMC11920621

[15] Siopi M, Peroukidou I, Beredaki M-I, Spruijtenburg B, de Groot T, Meis JF, Vrioni G, Tsakris A, Pournaras S, Meletiadis J. 2023. Overestimation of amphotericin B resistance in Candida auris with sensititre YeastOne antifungal susceptibility testing: a need for adjustment for correct interpretation. Microbiol Spectr 11:e0443122. doi:10.1128/spectrum.04431-22 37036351 PMC10269614

[16] da Silva KJG, Lucini F, Dos Santos RAC, Santos DA, Meis JF, Melhem M de SC, Peres NT de A, Bastos RW, Rossato L. 2025. How does antifungal resistance vary in Candida (Candidozyma) auris and its clades? Quantitative and qualitative analyses and their clinical implications. Clin Microbiol Infect 31:1146–1156. doi:10.1016/j.cmi.2025.04.003 40216246

[17] CLSI. 2022. Epidemiological Cutoff Values for Antifungal Susceptibility Testing. In CLSI supplement M57S, 4th ed. Clinical and Laboratory Standards Institute.

[18] Berkow EL, Lockhart SR, Ostrosky-Zeichner L. 2020. Antifungal susceptibility testing: current approaches. Clin Microbiol Rev 33:e00069–19. doi:10.1128/CMR.00069-19 32349998 PMC7194854

[19] 2024. EUCAST: warning against the use of AST devices for amphotericin B testing of Candida auris. Available from: https://www.eucast.org/fileadmin/src/media/PDFs/EUCAST_files/Warnings/Warnings_docs/EUCAST_AFST_Warning_regarding_Amb_test_against_C_auris_Final_19-04-2024_CG.pdf

[20] CLSI. 2022. AST news update June 2022: hot topic Candida auris update: method variability with amphotericin B susceptibility testing. Available from: https://clsi.org/about/blog/ast-news-update-june-2022-hot-topic

[21] CDC. 2024. Clinical Treatment of C. auris infections. https://www.cdc.gov/candida-auris/hcp/clinical-care/index.html.

[22] Siopi M, Leventaki S, Pachoulis I, Spruijtenburg B, Meis JF, Pournaras S, Vrioni G, Tsakris A, Meletiadis J. 2025. Evaluation of the MIC test strips for antifungal susceptibility testing of Candidozyma auris (Candida auris) using a representative international collection of isolates. J Clin Microbiol 63:e00399-25. doi:10.1128/jcm.00399-25 40608317 PMC12345157

[23] Arendrup MC, Lockhart SR, Wiederhold N. 2025. Candida auris MIC testing by EUCAST and clinical and laboratory standards institute broth microdilution, and gradient diffusion strips; to be or not to be amphotericin B resistant? Clin Microbiol Infect 31:108–112. doi:10.1016/j.cmi.2024.10.010 39426481 PMC11931498

[24] Siopi M, Pachoulis I, Leventaki S, Spruijtenburg B, Meis JF, Pournaras S, Vrioni G, Tsakris A, Meletiadis J. 2024. Evaluation of the Vitek 2 system for antifungal susceptibility testing of Candida auris using a representative international panel of clinical isolates: overestimation of amphotericin B resistance and underestimation of fluconazole resistance. J Clin Microbiol 62:e0152823. doi:10.1128/jcm.01528-23 38501836 PMC11005389

[25] Mantzana P, Protonotariou E, Meletis G, Tychala A, Skoura L. 2024. The Micronaut-AM antifungal susceptibility testing method does not overestimate amphotericin B resistance in Candida auris Microbiol Spectr 12:e0049024. doi:10.1128/spectrum.00490-24 38578100 PMC11064516

[26] Asadzadeh M, Ahmad S, Alfouzan W, Al-Obaid I, Spruijtenburg B, Meijer EFJ, Meis JF, Mokaddas E. 2024. Evaluation of etest and MICRONAUT-AM assay for antifungal susceptibility testing of Candida auris: underestimation of fluconazole resistance by MICRONAUT-AM and overestimation of amphotericin B resistance by etest. Antibiotics (Basel) 13:840. doi:10.3390/antibiotics13090840 39335013 PMC11428412

[27] Cantón E, Pemán J, Hervás D, Iñiguez C, Navarro D, Echeverría J, Martínez-Alarcón J, Fontanals D, Gomila-Sard B, Buendía B, Torroba L, Ayats J, Bratos A, Sánchez-Reus F, Fernández-Natal I, FUNGEMYCA Study Group. 2012. Comparison of three statistical methods for establishing tentative wild-type population and epidemiological cutoff values for echinocandins, amphotericin B, flucytosine, and six Candida species as determined by the colorimetric Sensititre YeastOne method. J Clin Microbiol 50:3921–3926. doi:10.1128/JCM.01730-12 23015676 PMC3503000

[28] Massic L, Doorley LA, Jones SJ, Richardson I, Siao DD, Siao L, Dykema P, Hua C, Schneider E, Cuomo CA, Rogers PD, Van HS, Parker JE, Kelly SL, Hess D, Rybak JM, Pandori M. 2025. Acquired amphotericin B resistance attributed to a mutated ERG3 in Candidozyma auris. Antimicrob Agents Chemother 69:e00601-25. doi:10.1128/aac.00601-25 PMC12587534 40980913

[29] Rybak JM, Barker KS, Muñoz JF, Parker JE, Ahmad S, Mokaddas E, Abdullah A, Elhagracy RS, Kelly SL, Cuomo CA, Rogers PD. 2022. In vivo emergence of high-level resistance during treatment reveals the first identified mechanism of amphotericin B resistance in Candida auris. Clin Microbiol Infect 28:838–843. doi:10.1016/j.cmi.2021.11.024 34915074 PMC9467277

[30] Lockhart SR. 2019. Candida auris and multidrug resistance: defining the new normal. Fungal Genet Biol 131:103243. doi:10.1016/j.fgb.2019.103243 31228646 PMC12012538

[31] Maphanga TG, Naicker SD, Kwenda S, Muñoz JF, van Schalkwyk E, Wadula J, Nana T, Ismail A, Coetzee J, Govind C, Mtshali PS, Mpembe RS, Govender NP, for GERMS-SA. 2021. In vitro antifungal resistance of Candida auris isolates from bloodstream infections, South Africa. Antimicrob Agents Chemother 65:e0051721. doi:10.1128/AAC.00517-21 34228535 PMC8370198

[32] O’Brien B, Liang J, Chaturvedi S, Jacobs JL, Chaturvedi V. 2020. Pan-resistant Candida auris: New York subcluster susceptible to antifungal combinations. Lancet Microbe 1:e193–e194. doi:10.1016/S2666-5247(20)30090-2 35544199

[33] Rhodes J, Abdolrasouli A, Farrer RA, Cuomo CA, Aanensen DM, Armstrong-James D, Fisher MC, Schelenz S. 2018. Genomic epidemiology of the UK outbreak of the emerging human fungal pathogen Candida auris. Emerg Microbes Infect 7:43. doi:10.1038/s41426-018-0045-x 29593275 PMC5874254

[34] Kordalewska M, Lee A, Park S, Berrio I, Chowdhary A, Zhao Y, Perlin DS. 2018. Understanding Echinocandin resistance in the emerging pathogen Candida auris. Antimicrob Agents Chemother 62:e00238-18. doi:10.1128/AAC.00238-18 29632013 PMC5971591

[35] Sharma D, Paul RA, Rudramurthy SM, Kashyap N, Bhattacharya S, Soman R, Shankarnarayan SA, Chavan D, Singh S, Das P, Kaur H, Ghosh AK, Prasad R, Sanyal K, Chakrabarti A. 2022. Impact of FKS1 genotype on echinocandin In vitro susceptibility in Candida auris and in vivo response in a murine model of infection. Antimicrob Agents Chemother; 67:e01243-22. doi:10.1128/AAC.01652-21 PMC8765266 34780273

[36] Espinel-Ingroff A, Arendrup MC, Pfaller MA, Bonfietti LX, Bustamante B, Canton E, Chryssanthou E, Cuenca-Estrella M, Dannaoui E, Fothergill A, Fuller J, Gaustad P, Gonzalez GM, Guarro J, Lass-Flörl C, Lockhart SR, Meis JF, Moore CB, Ostrosky-Zeichner L, Pelaez T, Pukinskas SRBS, St-Germain G, Szeszs MW, Turnidge J. 2013. Interlaboratory variability of Caspofungin MICs for Candida spp. using CLSI and EUCAST methods: should the clinical laboratory be testing this agent? Antimicrob Agents Chemother 57:5836–5842. doi:10.1128/AAC.01519-13 24018263 PMC3837874

[37] Espinel-Ingroff A, Alvarez-Fernandez M, Cantón E, Carver PL, Chen SC-A, Eschenauer G, Getsinger DL, Gonzalez GM, Govender NP, Grancini A, et al.. 2015. Multicenter study of epidemiological cutoff values and detection of resistance in Candida spp. to anidulafungin, caspofungin, and micafungin using the Sensititre YeastOne colorimetric method. Antimicrob Agents Chemother 59:6725–6732. doi:10.1128/AAC.01250-15 26282428 PMC4604361

[38] Oganesyan E, Klimenteva V, Vybornova I, Venchakova V, Parshikova E, Kovyrshin S, Orlova O, Kruglov A, Gordeeva S, Vasilyeva N, Taraskina A. 2025. Population structure based on microsatellite length polymorphism, antifungal susceptibility profile, and enzymatic activity of Candida auris clinical isolates in Russia. JoF 11:35. doi:10.3390/jof11010035 39852454 PMC11766443

[39] Lee WG, Shin JH, Uh Y, Kang MG, Kim SH, Park KH, Jang HC. 2011. First three reported cases of nosocomial fungemia caused by Candida auris. J Clin Microbiol 49:3139–3142. doi:10.1128/JCM.00319-11 21715586 PMC3165631

[40] Ceballos-Garzon A, Garcia-Effron G, Cordoba S, Rodriguez JY, Alvarez-Moreno C, Le Pape P, Parra-Giraldo CM, Morales-López S. 2022. Head-to-head comparison of CLSI, EUCAST, Etest and VITEK2 results for Candida auris susceptibility testing. Int J Antimicrob Agents 59:106558. doi:10.1016/j.ijantimicag.2022.106558 35227828

[41] Aldejohann AM, Wiese-Posselt M, Gastmeier P, Kurzai O. 2022. Expert recommendations for prevention and management of Candida auris transmission. Mycoses 65:590–598. doi:10.1111/myc.13445 35437832

[42] Kurt AF, Kuskucu MA, Balkan IiI, Baris A, Yazgan Z, Serife Oz A, Tosun AI, Mete B, Tabak F, Aygun G. 2021. Candida auris Fungemia and a local spread taken under control with infection control measures: First report from Turkey. Indian J Med Microbiol 39:228–230. doi:10.1016/j.ijmmb.2021.03.007 33785243

[43] de Groot T, Puts Y, Berrio I, Chowdhary A, Meis JF. 2020. Development of Candida auris short tandem repeat typing and its application to a global collection of isolates. mBio 11:e02971-19. doi:10.1128/mBio.02971-19 31911492 PMC6946803

[44] CLSI. 2017. Reference method for broth dilution antifungal susceptibility testing of yeasts. M27 4th ed. Clinical and Laboratory Standards Institute, Wayne, PA, USA.

